# Exploring power and power sharing in participatory health research partnerships: A scoping review protocol

**DOI:** 10.1371/journal.pone.0303799

**Published:** 2024-07-18

**Authors:** Nino Burduladze, Laundette P. Jones, Brian D. Jones, Uchizi Msowoya, Jon Salsberg, Anna Whitney, Meghan Gilfoyle

**Affiliations:** 1 School of Medicine, University of Limerick, Limerick, Ireland; 2 University of Maryland School of Medicine, Baltimore, MD, United States of America; 3 Community Consultant, The Prized Apex, Baltimore, Maryland, United States of America; 4 PPI Research Unit, School of Medicine, University of Limerick, Limerick, Ireland; 5 Health Research Institute, University of Limerick, Limerick, Ireland; 6 Department of Psychiatry, School of Medicine, Community Health Specialist, University of Maryland, Baltimore, MD, United States of America; Indian Institute of Public Health Gandhinagar, INDIA

## Abstract

**Introduction:**

Participatory health research (PHR) as a research paradigm, guides the research process and strives to achieve positive change in society in the interest of people’s health. In this scoping review, PHR will be used as an umbrella term considering a wide range of collaborative research approaches in the health context. PHR is conducted ’with’ or ’by’ those it intends to benefit, as opposed to ’on’ and ’for’ them. Their involvement throughout the research process seeks to shift power and decision-making from where they traditionally lay within academia toward community, patient and public end-users. Research cannot be truly participatory without concurrently addressing power dynamics within the partnership and power imbalances in decision making. Therefore, power sharing can be defined as a major factor in building effective academic-community collaborations. This scoping review aims to identify, clarify, and map existing literature on power and power sharing in PHR from both theoretical and practical perspectives. Specifically, we will explore how power is conceptualised throughout the literature, and how power and power sharing are applied and addressed in real-life PHR partnerships.

**Materials and methods:**

This scoping review will be conducted in accordance with the guidelines outlined in the Joanna Briggs Institute (JBI) Reviewer’s Manual. This scoping review will consider both empirical and non-empirical research that report on understanding power and power sharing in participatory health research partnerships. All appropriate studies will be retrieved from the following five electronic databases: PubMed, Scopus, Embase, PsycINFO, SocIndex. This review will be limited to articles published in English and from January 1998 to March 2024. As the scoping review aims to capture more than peer-reviewed and published literature, it will also include grey literature such as theses and dissertations, reports, conference proceedings, and editorials. Data from the included literature will be extracted based on the data extraction tool, defined in advance.

**Ethics and dissemination:**

As primary data will not be collected, ethical approval is not required to conduct the scoping review. The findings of this study will be disseminated through peer-reviewed publications.

## Introduction

### Background

Participatory Health Research (PHR) is discussed as a “systematic inquiry” entailing the active involvement of the individuals impacted by the issue being studied, for the purposes of learning and taking action or effecting change [1]. It can be seen as an umbrella term that encompasses a range of research approaches [2, 3], such as, co-creation, collaborative knowledge generation by academics working alongside other stakeholder [4]; integrated knowledge translation (IKT), a participatory approach that primarily partners with clinicians, service managers and policy makers, as well as patients and other end-users [4]; public and patient involvement which focuses predominantly on the involvement of patients and their carers [5]; and community-based participatory research (CBPR) that typically focuses on the involvement of communities and community-based stakeholders to address a wide range of problems ranging from individual care up to public health policy intervention [6]. Regardless of the term used, all share a philosophy of inclusivity and end-user involvement throughout the research process ensuring that those who the research intends to benefit are at the heart of the research decision-making process [7].

The defining principle of PHR is to maximise the involvement of those whose life or work is the subject of research in all stages of the research process. PHR strives to harness equitable opportunity for all research partners (i.e., those with both experiential and methodological expertise) to be meaningfully involved in the research process to the greatest extent possible [8], while intentionally creating collaborative spaces [9] between those who typically have power and those that often do not (i.e., doctors together with patients, policy makers together with refugees, or academic researchers with community partners). Participatory processes help ensure ‘transparent involvement of different stakeholders’ [10] and that all voices are heard. Thus, through these processes, PHR enables a shift in power and decision-making from where they traditionally lay within academia towards community, patient and public end-users [11].

Achieving this level of participation at all stages of health research is challenging and affected by social hierarchies, gender, power, privilege, marginalisation and other factors [12]. Power imbalances among academic researchers and non-academic participants, jeopardises the ability of PHR to bring about positive and long-term change [13]. Research can entail different degrees of participation, but formal research involvement that does not concurrently address power dynamics among partners and power imbalances in decision making is not truly participatory [14]. Thus, existing literature defines power sharing as a major factor in building effective academic-community collaborations [14]. Recognising the importance of power and power sharing, a group of researchers from the International Collaboration for Participatory Health Research (ICPHR) created online workshops to explore how PHR projects, from a variety of global contexts, understand the dimensions of power and power sharing [13]. Results from this qualitative study demonstrated that while participatory researchers acknowledge the importance of unpacking “power”, the concept is rarely discussed and addressed explicitly in research partnerships [13]. Thus, although commonly discussed in the literature as an imperative part of the PHR process, gaps in understanding the practical aspects of power and power sharing persist, both conceptually and operationally [3, 10, 11, 13].

### Objectives of scoping review

This scoping review aims to identify, clarify, and map existing literature on power and power sharing in PHR from both theoretical and practical perspectives. Specifically, we will explore how power is conceptualised throughout the literature, and how power and power sharing are operationalised in real-life participatory health research partnerships. In doing so, we will answer the following research question:

“How is power conceptualised, operationalised and measured both theoretically and practically in participatory health research partnerships?”

Accordingly, objectives of this scoping review are to:

1. Explore how power is conceptualised, operationalised and measured in PHR.
2. Determine whether existing literature discusses power in PHR and if so, at what stage? (i.e., who holds funds, decisions about methodology, who gets to be co-investigator, etc.).
3. Report on how (if at all) authors suggest addressing power and power inequities in PHR partnerships and the barriers and facilitators of this.
4. Explore if there are any contextual differences to the above objectives (i.e., geographical, cultural, socioeconomic, demographics (e.g., race/ethnicity, etc.)).

## Materials and methods

A scoping review was deemed appropriate for this research as it is considered as an effective tool for exploring, mapping and summarising the breadth of literature in a given field, when the scope of the review and research question are broad in focus [15]. This scoping review will be conducted in compliance with the most up-to-date scoping review guidelines outlined in the Joanna Briggs Institute (JBI) Reviewer’s Manual [16]. The JBI Reviewer’s Manual is premised on the guidelines established by Levac *et al*, [17], which is a further refined methodological framework from Arksey and O’Malley [18]. We will incorporate the Population (or participants), Concept and Context (PCC) framework [16] to identify these elements of interest for this scoping review.

### Inclusion criteria

Detailed justification for inclusion and exclusion criteria is outlined below in Table 1.

**Table 1:** Eligibility criteria.

Criterion	Inclusion	Exclusion	Justification
Population and Sample	Humans over 18	• Any study population other than humans, i.e. animal studies; • Humans under the age of 18 (e.g. children)	• Referring to understanding power in participatory health research partnerships conceptually and in real-life settings; • Different power dynamics to consider when referring to children compared to adults.• Different ethical considerations when involved people under the age of 18 in research [19].
Language	Written in English	Any other language that is not English	• Reviewers only speak English and thus can only confidently screen literature in English
Time Period	1998–2024	Outside this time period	• In 1996, the North American Primary Care Research Group (NAPCRG) held a preconference workshop called ’Responsible Research with Communities’. After that, the use of PR principles in health-related research became more common. Particularly, this workshop was followed by the adoption of a Policy Statement promoting participatory research later in 1998.
Study Focus	Articles that discuss about the concept of power/power sharing in participatory health research or community-based participatory research partnership or patient and public involvement. Articles that show how power or/and power sharing are conceptualised, operationalised or measured practically in participatory health research partnership.	• Must be participatory health research, not other forms of involvement outside of the health research context (i.e., co-management, shared decision-making about care, patient participation). • Literature focusing solely on empowerment.• Literature discussing about power which is operating at the macro level. For this purpose, the macro level is defined as socio-political structures such as historical factors, governance/rule of law, different population etc. [10]	• Scoping review attempts to identify, clarify, and map existing literature (if any) on power, and power sharing, in participatory health research from both theoretical and practical perspectives. In particular, how is power defined and conceptualised in existing literature, and how power sharing is applied and addressed in real-life participatory health research partnerships. • Empowerment was deemed conceptually different from power or power sharing [20]. • For this scoping review, the term ‘power’ is only considered in social context contrary to biological/chemical/engineering or related contexts. • As described by Roura et al., 2021 power dynamics operate within and across the individual (micro), interpersonal (meso), and structural (macro) levels. The current review is concerned with power dynamics at the micro level of individual values and attitudes and meso level of inter-sectorial spaces. The power dynamics at the macro level is very complex and includes both visible and invisible power hierarchies compared to the with power dynamics at the micro level which is mostly concerned with the values and attitudes of the individual [10]
Type of article	Peer reviewed journal articles or reviews and grey literature. Specifically, grey literature will include theses/dissertations, reports, conference proceedings, editorials and chapters in a textbook.	Any other literature that is not listed in the inclusion criteria, such as websites.	Scoping review aims to capture more than peer reviewed and published literature in order to expansively explore a broad research question. Preliminary searches of grey literature generally revealed those listed in our inclusion criteria Acknowledging feasibility and time constraints, we felt the literature criteria listed would be sufficient in capturing the necessary literature to inform our review and ultimately, a conceptual framework
Geographic Location	Any location–an international and global context.	None	The context of the review is PHR globally and internationally

### Types of participants

Only people over the age of 18 will be considered for the study’s sample. This is because there are different ethical considerations when involving those under the age of 18 in research [19]. The partnership may include individuals or groups of people who are participating in health research and, directly or indirectly, are affected by the results and findings of the research.

### Concept

The main concept under investigation is power and power sharing, which will be examined in four ways. First, we will explore how literature conceptualises power, and whether different types or levels of power exist (e.g., power within, power with, power to, power over) [21, 22]. The second aspect is surrounding how power is operationalised and measured among partners from both theoretical and practical perspectives, how partners perceive it, who oversees facilitating the process in a real-life setting, and how participants are engaged and involved in the PHR process. When we say theoretically, we mean identifying how authors would define the concept of power and power sharing as well its effects; while practically refers to how these theoretical insights are applied to sustain functioning research partnerships [23]. Third, we will explore if power inequities are discussed in the literature and if/how authors sought to address these inequities. Finally, we will explore how all the above differ across varying contexts (i.e., geographical, cultural, socioeconomic etc.).

### Context

The context of interest for this scoping review is PHR acting as an umbrella term for other types of health-related collaborative research approaches, in which all aspects of the abovementioned concept will be examined. Further, any other forms of participatory research outside of the health context will be excluded.

This review will be limited to articles published from January 1998 until March 2024. This timeframe reflects the 1998 adoption of the North American Primary Care Research Group’s (NAPCRG) Policy Statement on *Responsible Research with Communities*: *Participatory Research in Primary Care* [24] after which the use of PHR principles in health-related research became more common [24].

As the reviewers only speak English, studies in languages other than English will be excluded. Furthermore, the scoping review’s context is PHR globally and internationally, thus, no country or geographical location restrictions will be used during the screening process.

### Types of sources

This scoping review will consider all types of quantitative, qualitative, and mixed-methods studies and reviews that report on understanding power and/or power sharing in participatory health research partnerships, respectively, responding to the review objectives outlined above.

Grey literature such as theses and dissertation, unpublished articles, opinion pieces, reports, conference proceedings, would also be considered. Besides, systematic reviews that meet the inclusion criteria will also be considered.

### Search strategy

All aspects of the search strategy development, including choosing the appropriate databases, have been carried out in conjunction with a faculty librarian from the University of Maryland, Baltimore. To identify relevant studies and articles, five electronic databases will be searched, including PubMed, Scopus, Embase, PsycINFO, SocIndex. In addition, grey literature will be searched in Google Scholar and Open Grey databases.

Following the guidance of JBI [25], a three-step search strategy will be used. First, a preliminary search of PubMed, and Scopus will be conducted. This initial search will then be followed by an analysis of the text contained in the title, abstracts and index terms of retrieved papers. A second search using all identified keywords and index terms will be applied across all databases, including PubMed, Scopus, Embase, PsycINFO, SocIndex. Finally, we will review additional sources from the reference lists of the literature selected for full-text screening. The complete search strategy from the PubMed database can be found in S1 File.

### Study selection

Following the search, results from all databases will be exported to Covidence software [26] for screening. Before each source is screened for eligibility, duplicates will be removed. The screening process for study selection will be conducted by two independent reviewers in two stages. First, two independent reviewers will screen the literature by titles and abstracts based on the predetermined inclusion criteria. Following, all included articles from the title and abstract screening will be screened at full text, documenting at this stage, the reasons for the exclusion. If the two independent screeners disagree, the final decision will be made by a third reviewer/arbitrator. Following the guidance from JBI Reviewer’s Manual at each review stage, all reviewers (including arbitrator) will screen approximately 10% of the same literature. They will then meet with the entire research team to discuss their thought process, decision-making, and revisit (and refine if pertinent) the eligibility criteria. Reviewers will then repeat this step, and will only proceed independently once an agreement rate of 75% or greater is achieved [16].

### Data extraction

Two independent reviewers will extract data from the included literature using the data extraction tool based on Peters at al.,[16] and refined by the reviewers. Extracted information will include, for example, publication details, general study details, and key findings relevant to the review objectives. See Table 2 below for the preliminary charting table. If there are any changes required during the data extraction process (i.e., key findings related to the concept of power, additional study details etc.), a detailed description of such changes will be reported in the full scoping review. Preferred Reporting Items for Systematic Reviews and Meta-Analyses flow chart (PRISMA) (found in S2 File) [27] will be also used to identify the number of included and excluded articles at each stage of the review process.

**Table 2:** Preliminary table for data extraction and associated questions.

Extracted data	Associated question
**Publication details**	
Author(s)	Who wrote the study/document?
Year of publication	When was the study/document published? (year)
Origin/country of origin	Where was the study/document conducted and/or published?
Publication type	What type of publication is this? (Empirical study or grey literature)
**General study details**	
Aims/purpose	What were the aims of the study/document?
Methodological design	What methodological design was used for this study?
Study population and sample size (if applicable)	Who is the target population of the study and how many (n) were included in the study?
Methods	What specific methods were use in this study?
Intervention type, (if applicable)	Was an intervention used in this study?
Comparator and duration of the intervention (if applicable)	If yes to the intervention type, what was the comparator and duration of the intervention?
Outcomes and details of these (if applicable)	What was the study outcome?
**Key findings that relate specifically to the concept of power**	
What is the context of power?	Is the study/document conceptualising or operationalising power in PHR?
How power is conceptualised?	How does the study define power?
How power is operationalised? (Theoretically and practically)	What are the dimensions and indicators used for power? What operationalisation issues exist? What observations were made? Is any mechanism defined to facilitate the research process in a real-life setting?
How power is measured?	What level of measurement was used (nominal, ordinal, interval, ratio) to measure power? What type of measures was (survey, scaling, qualitative, unobtrusive) used for power?
How (if any) do authors suggest addressing power and power inequities in PHR partnership? (Theoretically and practically)	What kind of theoretical and/or practical methods are defined addressing balanced power sharing in PHR partnership?

### Data analysis and presentation

The extracted studies will then be analysed using frequencies and counts, providing a narrative summary of the findings based on our research objectives. Our findings will be discussed collaboratively by a team of knowledge users and experts in relation to gaps in the literature and future implications. The findings will contribute to the development of a framework for assessing the level of power sharing attained in PHR projects and practical guidelines.

### Consultation with knowledge users

In the advanced and extended methodology of the scoping review [25], an additional stage of expert consultation (e.g. with knowledge users or experts by experience) was suggested. The present scoping review will be conducted in collaboration with two institutions (the University of Limerick and the University of Maryland, Baltimore) and a community partner providing expert knowledge by experience, (co-author BJ) residing in Baltimore, Maryland, USA. The rationale for this scoping review was derived from a previous workshop by Egid et al., (discussed above), capturing the experiences of participatory health researchers in relation to power. Findings from the workshop inspired future research in this field to gain a better understanding of the concept of power and power sharing in PHR partnerships. Afterwards, these insights will be discussed with the group of researchers from the University of Maryland, Baltimore and the community partners from the surrounding community. The detailed format for structured stakeholder discussion will be considered later. We anticipate that gaining the perspectives from non-academic stakeholders will contribute to a more complete understanding of power inequities and the co-production of knowledge in PHR.

## Supporting information

S1 Checklist PRISMA-P (Preferred Reporting Items for Systematic review and Meta-Analysis Protocols) 2015 checklist: Recommended items to address in a systematic review protocol*. (DOCX)

S1 File Search strategy. (DOCX)

S2 File PRISMA flow chart 2020. (DOCX)

## References

[1] Green LW, Canada RSo, Research BCfHP, editors. Study of participatory research in health promotion: Review and recommendations for the development of participatory research in health promotion in Canada1995: Royal Society of Canada

[2] Salsberg J, Parry D, Pluye P, Macridis S, Herbert CP, Macaulay AC. Successful Strategies to Engage Research Partners for Translating Evidence into Action in Community Health: A Critical Review. Journal of Environmental and Public Health. 2015;2015:191856. doi: 10.1155/2015/191856 25815016 PMC4359847

[3] Cargo M, Mercer SL. The Value and Challenges of Participatory Research: Strengthening Its Practice. Annual Review of Public Health. 2008;29(1):325–50. doi: 10.1146/annurev.publhealth.29.091307.083824 18173388

[4] Nguyen T, Graham ID, Mrklas KJ, Bowen S, Cargo M, Estabrooks CA, et al. How does integrated knowledge translation (IKT) compare to other collaborative research approaches to generating and translating knowledge? Learning from experts in the field. Health research policy and systems. 2020;18(1):35–. doi: 10.1186/s12961-020-0539-6 32228692 PMC7106699

[5] Wilson P, Mathie E, Keenan J, McNeilly E, Goodman C, Howe A, et al. ReseArch with Patient and Public invOlvement: a realisT evaluation: the RAPPORT study. Health services and delivery research. 2015. 26378332

[6] Israel BA, Schulz AJ, Parker EA, Becker AB. Review of community-based research: assessing partnership approaches to improve public health. Annu Rev Public Health. 1998;19:173–202. doi: 10.1146/annurev.publhealth.19.1.173 9611617

[7] Salsberg. Using Social Network Analysis to Measure Community Ownership in Participatory Health Research. London 2020. Available from: https://methods.sagepub.com/case/social-network-analysis-community-ownership-participatory-health-research.

[8] (ICPHR) ICfPHR. Position Paper 2: Participatory Health Research: A Guide to Ethical Principals and Practice. 2013(Berlin).

[9] MacFarlane A, Dowrick C, Gravenhorst K, O’Reilly-de Brún M, de Brún T, van den Muijsenbergh M, et al. Involving migrants in the adaptation of primary care services in a ‘newly’ diverse urban area in Ireland: The tension between agency and structure. Health & Place. 2021;70:102556. 34214893 10.1016/j.healthplace.2021.102556

[10] Roura M. The Social Ecology of Power in Participatory Health Research. Qual Health Res. 2021;31(4):778–88. doi: 10.1177/1049732320979187 33349170

[11] Salsberg J, Macridis S, Garcia Bengoechea E, Macaulay AC, Moore S. The shifting dynamics of social roles and project ownership over the lifecycle of a community-based participatory research project. Fam Pract. 2017;34(3):305–12. doi: 10.1093/fampra/cmx006 28334748

[12] Fielden SJ, Rusch ML, Masinda MT, Sands J, Frankish J, Evoy B. Key considerations for logic model development in research partnerships: A Canadian case study. Evaluation and Program Planning. 2007;30(2):115–24. doi: 10.1016/j.evalprogplan.2007.01.002 17689318

[13] Egid BR, Roura M, Aktar B, Amegee Quach J, Chumo I, Dias S, et al. ’You want to deal with power while riding on power’: global perspectives on power in participatory health research and co-production approaches. BMJ Glob Health. 2021;6(11). doi: 10.1136/bmjgh-2021-006978 34764147 PMC8587355

[14] Macaulay AC, Commanda LE, Freeman WL, Gibson N, McCabe ML, Robbins CM, et al. Participatory research maximises community and lay involvement. North American Primary Care Research Group. Bmj. 1999;319(7212):774–8. doi: 10.1136/bmj.319.7212.774 10488012 PMC1116604

[15] Tricco AC, Lillie E, Zarin W, O’Brien K, Colquhoun H, Kastner M, et al. A scoping review on the conduct and reporting of scoping reviews. BMC Medical Research Methodology. 2016;16(1):15. doi: 10.1186/s12874-016-0116-4 26857112 PMC4746911

[16] Peters MD, Marnie C, Tricco AC, Pollock D, Munn Z, Alexander L, et al. Updated methodological guidance for the conduct of scoping reviews. JBI evidence synthesis. 2020;18(10):2119–26. doi: 10.11124/JBIES-20-00167 33038124

[17] Levac D, Colquhoun H, O’Brien KK. Scoping studies: advancing the methodology. Implementation Science. 2010;5(1):69. doi: 10.1186/1748-5908-5-69 20854677 PMC2954944

[18] Arksey H O’Malley L. Scoping studies: towards a methodological framework. International Journal of Social Research Methodology. 2005;8(1):19–32.

[19] Koskinas E, Gilfoyle M, Salsberg J. Exploring how patients, carers and members of the public are recruited to advisory boards, groups and panels as partners in public and patient involved health research: a scoping review protocol. BMJ Open. 2022;12(4):e059048. doi: 10.1136/bmjopen-2021-059048 35450910 PMC9024234

[20] Research ICfPH. Empowerment and Participatory Health Research. Position Paper No. 5. 2021.

[21] Popay J, Whitehead M, Ponsford R, Egan M, Mead R. Power, control, communities and health inequalities I: theories, concepts and analytical frameworks. Health Promotion International. 2020;36(5):1253–63. 10.1093/heapro/daaa133 PMC8515177 33382890

[22] Powell K, Barnes A, Anderson de Cuevas R, Bambra C, Halliday E, Lewis S, et al. Power, control, communities and health inequalities III: participatory spaces—an English case. Health promotion international. 2021;36(5):1264–74. doi: 10.1093/heapro/daaa059 33382870 PMC8515175

[23] MacFarlane AE, Salsberg J. Conceptualising and theorising for research co-production. In: Graham ID. R-MJ, Kothari A., McCutcheon C., editor. Research Coproduction in Healthcare. Hoboken, NJ: Wiley-Blackwell 2022.

[24] Macaulay AC. Participatory research: What is the history? Has the purpose changed? Family Practice. 2016;34(3):256–8. 10.1093/fampra/cmw117 27993909

[25] Peters MDJ GC, McInerney P. Chapter 11: scoping reviews. In: Adelaide A, The Joanna Briggs Institute, editor. https://synthesismanual.jbi.global/2017.

[26] Covidence systematic review software, Veritas Health Innovation, Melbourne, Australia. Available at www.covidence.org. [Internet]. 2022. Available from: www.covidence.org.

[27] Page MJ, McKenzie JE, Bossuyt PM, Boutron I, Hoffmann TC, Mulrow CD, et al. The PRISMA 2020 statement: an updated guideline for reporting systematic reviews. BMJ. 2021;372:n71. doi: 10.1136/bmj.n71 33782057 PMC8005924

