# Supplementary material for: Exploring power and power sharing in participatory health research partnerships: A scoping review protocol
Source: PLoS One. 2024 Jul 18;19(7):e0303799. doi: 10.1371/journal.pone.0303799 (PMC11257268; doi:10.1371/journal.pone.0303799)
Supplement: S1 File — (DOCX) [file pone.0303799.s002.docx]

### **Appendix I: Search Strategy**

Search strategy for PubMed conducted on 05-APR-2022

**Filters:** from 1998-present, Language - English

**Results:** 1752 studies were retrieved.

((((CBPR[Title/Abstract] OR action research[Title/Abstract] OR participative research[Title/Abstract] OR participatory research[Title/Abstract] OR co-production[Title/Abstract] OR co-researcher* OR research partnership[Title/Abstract] OR emancipator research[Title/Abstract] OR participatory rural[Title/Abstract] OR collaborative inquiry[Title/Abstract] OR decolonizing methodolog*[Title/Abstract] OR appreciative inquiry[Title/Abstract] OR dialectic* inquiry[Title/Abstract] OR cooperative inquiry[Title/Abstract] OR community-partnered action research[Title/Abstract] OR community-driven action research[Title/Abstract] OR community-driven research[Title/Abstract] OR participatory evaluation[Title/Abstract] OR community engaged research[Title/Abstract] OR community-university[Title/Abstract] OR community-academic[Title/Abstract] OR collective knowledge[Title/Abstract]) OR ("Consumer Advocacy"[Mesh] AND research)) OR ("Community-Institutional Relations"[Mesh])) OR ("Community-Based Participatory Research"[Mesh])) AND ("Power, Psychological"[Mesh] OR ("power"[Title/Abstract] OR "resource sharing"[Title/Abstract] OR "empowerment"[Title/Abstract] OR "resource allocation"[Title/Abstract] OR "shared decision making"[Title/Abstract] OR "social capital"[Title/Abstract] OR "epistemic injustice"[Title/Abstract] OR "silencing"[Title/Abstract]))
