# Supplementary material for: Exploring power and power sharing in participatory health research partnerships: A scoping review protocol
Source: PLoS One. 2024 Jul 18;19(7):e0303799. doi: 10.1371/journal.pone.0303799 (PMC11257268; doi:10.1371/journal.pone.0303799)
Supplement: S2 File — (DOCX) [file pone.0303799.s003.docx]

### **Appendix II. PRISMA Flow Chart 2020**

**Identification of studies via databases and registers**

Records removed *before screening*:

Duplicate records removed (n = )

Records marked as ineligible by automation tools (n = )

Records removed for other reasons (n = )

Records identified from*:

Databases (n = )

Registers (n = )

**Identification**

Records screened

(n = )

Records excluded**

(n = )

Reports sought for retrieval

(n = )

Reports not retrieved

(n = )

**Screening**

Reports assessed for eligibility

(n = )

Reports excluded:

Reason 1 (n = )

Reason 2 (n = )

Reason 3 (n = )

etc.

Studies included in review

(n = )

Reports of included studies

(n = )

**Included**
