# Supplementary material for: Exploring power and power sharing in participatory health research partnerships: A scoping review protocol
Source: PLoS One. 2024 Jul 18;19(7):e0303799. doi: 10.1371/journal.pone.0303799 (PMC11257268; doi:10.1371/journal.pone.0303799)
Supplement: Attachment — Submitted filename: Responses to Reviewers.docx [file pone.0303799.s004.docx]

Dear Reviewers,

We appreciate your suggestions. The authors have incorporated the necessary changes and explanations into our manuscript based on your suggestions.

Please refer to Table 1 below for the amendments made, where appropriate.

Table #1 – Responses to the reviewer.

| **Recommendations** | **Amendments (if any)** | **Comments from the authors** | |
| --- | --- | --- | --- |
| suggestion a revision of the title by removing ‘moving from theory to practice’. It is very wordy and loses its’ impact | *Exploring power and power sharing in participatory health research partnerships: a scoping review protocol* | Amended as requested | |
| Typo line 39 – how power is? | *We will explore how power is conceptualised throughout the literature* | Amended as requested | |
| Query data limiter to April 2022? Is there a justification for excluding literature published from April 2022 to present? Introduction or background – clarify? | Changes have been applied to line 48 | By the time when we first submitted this protocol, including the literature as of April 2022 seemed recent and relevant. Given the current circumstances, searches will be expanded to March, 2024. | |
| I would argue PHR is not just about generating new knowledge (lines 61-62) but also about challenging existing knowledge, structures, processes etc. I think this is a very narrow understanding of PHR. The definition here is more participatory action research which is an approach of itself. I note this is not mentioned in the following lines. | Changes have been applied to lines 61-64 | Thanks for your contribution, we have discussed and decided to include the definition of PHR by Green LW, which is broadly accepted in various literature | |
| There is no mention of co-design being under the umbrella of PHR? This is quite topical at present, particularly for health services research. | Changes have been applied to lines 65-66 | Thanks for your comment, we have amended and used the term “co-creation” which encompasses co-design | |
| Lines 81 onwards – I would argue that research funding (competitive grants), timelines, and institutions also contribute to power imbalances (structures) not just people (i.e. researchers). | No further amendments have been applied | As described by Roura et al., 2021 power dynamics operate within and across the individual (micro), interpersonal (meso), and structural (macro) levels. The current review is concerned with power dynamics at the micro level of individual values and attitudes and meso level of inter-sectorial spaces. The power dynamics at the macro level is very complex and includes both visible and invisible power hierarchies compared to the with power dynamics at the micro level which is mostly concerned with the values and attitudes of the individual. This is reflected in Study Focus of Table 1. | |
| Before the objectives of the scoping review are presented, it would be useful to provide a stanza highlighting knowledge gaps in existing reviews, whether PROSPERO and JBI were searched for existing or proposed reviews that are similar, and a clear justification for undertaking a scoping review on this topic area in the context of what is and is not published. | N/A | The librarian from the University of Maryland, Baltimore, has conducted a preliminary search since April 2022 – up to day, to identify existence of any other systematic or scoping review in respect of same topic. Therefor, it has been confirmed that there is no published reviews covering the context of power in PHR. | |
| It would be useful to expand on the justification for focusing only on adult populations, particularly given children and young people are vulnerable groups. | No further amendments have been applied | We recognise that children and youth constitute a significant focus of participatory research, particularly with respect to power and vulnerability. However, they constitute a special case with additional ethical considerations that would stretch beyond the scope of this current review, and deserve a review of its own. Therefor for pragmatic reasons, we decide to limit our focus to individuals over 18. | |
| Please justify limitation to 2022. This implies to me that the review has possibly been undertaken already in which case it is pointless to peer-review a protocol that may require changes to the search strategy employed. Please clarify. | Has been revised | This has been expanded to March 2024, please see the comment above | |
| Studies that report on patient and public involvement are not necessarily PHR – particularly given many journals require a section on patient and public involvement as part of the methods section or as a separate section. Please clarify how you will differentiate between PPI or PHR, and PHR which includes PPI. | No further amendments have been applied | Thank you for this comment, and we do recognise that not all PPI is PHR. However, for the purposes of this review, we are including studies where patient or public involvement meets certain minimum criteria to be considered participatory. Ie., that PPI contributors are involved in some capacity in at least the areas of contributing to the goals and design of the study, discussing and interpreting the results, or disseminating the findings. | |
| Geographic location – given the broad scope of the review and potential ambiguity in definitions of power in studies attributed to context and language, it may be worth considering narrowing the review by geographical setting (e.g., high income countries, middle-income countries, low-income countries using OECD criteria) as there will be variations in issues of power according to geographical setting. | No further amendments have been applied | Although there are certainly nuances to be considered between high-, middle- and low-income countries, or the geographical locations (north and south), we find that virtually all of the identified power issues are universally distributed. Therefore, adding a geographic criteria would limit more than it would add. | |
| Types of participants – please expand on the different ethical issues for those aged under 18 years. | No further amendments have been applied | Addition to above comments, all jurisdictions provide for additional ethical protection to minors above and beyond those provided to other vulnerable populations. Therefore, research undertaken on this segment of the population tends to apply means of addressing power and governance that differ considerably from research involving adults, even those from vulnerable populations. We feel that this is different enough to warrant being an exclusion criteria; and deserves its own treatment in a separate review. | |
| Concept – it would be useful to define what ‘power’ is in terms of the scope of this review, or at least what power is not. I feel this could be quite subjective and create a lot of conflicts in the screening process. | No further amendments have been applied | Thank you for this comment. And although your point is well taken, we are extremely hesitant to offer any definitions of power, or even the scope of its conceptualisation beyond what we have set out. We feel this may bias the selection and retention of items. This is a 'scoping' review, and our intent is to cast as wide a net as possible over the concept of power. | |
| Search Strategy - I assume step one of the JBI three-step search strategy has been undertaken given Appendix 1. If so, please write in past tense. | The changes have been applied to lines 172-173 | | N/A |
| Data analysis and presentation - Will a quality appraisal of studies be undertaken | N/A | | We appreciate your suggestion, but our decision is not to include that phase in this scoping review, especially when it's is only an optional stage as per JBI Manual. |
| Search results are very narrow for the types of keywords and MESH terms used. Has the search strategy been reviewed by a librarian as per the protocol | N/A | | The number of search results is lower because keywords were limited to title/abstract, in addition to the MeSH terms. This decision was made because without the title/abstract restrictions, the number of results in PubMed alone was over 100,000. We can also confirm, that the search strategy has been created by the faculty librarian from University of Maryland, Baltimore. |
| There are a few typos in the search strategy e.g., ‘emancipator’ should be emancipator*? Or even better, emancipat* to capture all truncations. Other keywords should be truncated too e.g., participative research to participat* res* to capture all possibilities. Proximity searches should be used too. | The relevant change is applied to line 324 | | Emacipator* is a typo and has been corrected, please note that this will be reflected in the following search. Other truncations as listed here would not have changed the search results as we captured the two versions of "participat*" that we wanted with "participative" and "participatory". Also, it is not possible to truncate words down to 3 characters in PubMed- the minimum is 4 characters, so res* is not feasible option.  Proximity operators could be used in some of these phrases instead of exact phrase (which we aim to use) but not all the databases we have define for our searches, include proximity operators, so the searches will not be parallel across all databases. |
| Further, the search strategy should be structured around the ‘PCC’. Currently there is no reference to what a ‘health context’ is in the search strategy or elsewhere in the protocol. Please consider. | N/A | | Including something as broad as "health" in a search strategy, especially something run in Pubmed or Embase, doesn't really help in any way and can be detrimental if any keywords are missed, like mentioning a specific rare disease or something where the abstract doesn't have anything broader in it. The manual screening process, ensures that health context is considered conceptually instead of just by keywords. |

Kind Regards,

Nino Burduladze

29/04/2024
